# In silico workflow for validation of patient-specific 3D-printed casts in forearm fracture immobilization

**DOI:** 10.3389/fsurg.2026.1765652

**Published:** 2026-03-23

**Authors:** Marton Bartos, Agoston Jakab Pokorni, Benjamin Hajnal, Mate Turbucz, Laszlo Horvath-Szekely, Karsa Ferenc Molnár, Nándor Orosz, Sebastian Frederick Bigdon, Peter Endre Eltes

**Affiliations:** 1 School of PhD Studies, Semmelweis University, Budapest, Hungary; 2 In Silico Biomechanics Laboratory, National Center for Spinal Disorders, Buda Health Center, Budapest, Hungary; 3 Department of Orthopaedic Surgery and Traumatology, Inselspital, University Hospital, University of Bern, Bern, Switzerland; 4 National Center for Spinal Disorders, Buda Health Center, Budapest, Hungary; 5 Department of Spine Surgery, Department of Orthopaedics, Semmelweis University, Budapest, Hungary

**Keywords:** 3D-printed, biomechanics, finite element analysis, forearm fracture, patient-specific cast

## Abstract

**Background:**

This paper aims to establish an in silico workflow for biomechanical assessment of patient-specific 3D-printed forearm casts using finite element analysis, and to apply this workflow to compare a novel computationally designed point-of-care cast with a commercially available state-of-the-art cast.

**Methods:**

A finite element model of a human forearm was generated from CT data, incorporating bones, soft tissues, and a simulated distal radius and ulna fracture. Two cast designs were virtually applied: a commercial cast (AA_CAST) and a point-of-care cast (POC_CAST) featuring a hexagonal lattice. The POC_CAST was simulated with three 3D-printing materials: Acrylonitrile Butadiene Styrene, Resin, and Polyamide. Six physiological loading conditions were assessed with the proximal end constrained: flexion, extension, radial and ulnar deviation (400 N load), and pronation and supination (1 Nm moment). Maximum von Mises stress in the casts and at the fracture surfaces, as well as maximum fracture displacement, were evaluated.

**Results:**

The AA_CAST model demonstrated superior fracture stabilization, showing consistently lower maximum von Mises stresses and displacements across all loading conditions. The POC_CAST exhibited its highest internal stresses during extension (36–37 MPa) and the largest fracture displacements during radial deviation (0.28–0.36 mm). In the POC_CAST simulations, printing material influenced fracture displacement but had negligible effect on maximum cast stress. All simulated configurations maintained fracture displacement below 0.4 mm, indicating adequate immobilization performance.

**Conclusion:**

The in silico workflow proved effective for biomechanical evaluation of patient-specific 3D-printed casts and enabled direct comparison between a novel POC design and a commercial standard. While the commercial cast provided superior stabilization in simulation, the POC_CAST also demonstrated mechanically sound performance. These findings support the workflow as a robust tool for preclinical assessment, iterative design, and material selection for orthopedic devices.

## 1 Introduction

With the advancement of three-dimensional (3D) printing technology (1), personalized medicine (2), particularly for orthopedic applications (3) has been revolutionized. One of the most distinguished inventions is the development of patient-specific casts for the immobilization of forearm fractures (4). In a clinical trial with 60 patients (5), the 3D-printed cast group showed higher scores in clinical efficacy, wrist function, and patient satisfaction, indicating its potential advantages in orthopedic treatment over conventional methods. In order to successfully apply 3D-printed casts, thorough validation is, however, critical; such validation ensures the casts have the necessary mechanical soundness, biocompatibility, as well as general clinical performance (4). In silico modeling and simulation are an incredibly robust paradigm for the validation of the performance of these innovatively designed orthopedic devices prior to the actual physical construction and operation (6). Finite element models of 3D printed casts can be used to predict their biomechanical behavior using computational analysis. Load conditions can be applied to different points in the model, which allows the identification of stress concentration points. The design can be further optimized regarding geometrical configuration to avoid these weak points. A better understanding of the mechanical properties will allow for the implementation of lattice geometry at identified locations and the use of the ideal infill density. In addition to evaluating structural integrity, computation can be implemented into the additive manufacturing process itself to ensure appropriate layer construction throughout the whole build. The resulting casts are then both light and mechanically strong. The integration of additive manufacturing into orthosis fabrication provides a cost-effective alternative to the traditional method (7, 8). A distal radius fracture is the most common fracture in the forearm (9). It is usually caused by falling on an outstretched hand. It accounts for the majority of upper extremity fractures. An ulnar fracture alone is uncommon but can be associated with a distal radius fracture. Radius and ulna fractures occur in a bimodal pattern, affecting younger patients following high-energy trauma and elderly patients after low-energy falls (10). Treatment is tailored to the specific injury pattern, with surgical fixation required for complex cases and conservative care employed for stable fractures. Forearm fractures are initially managed with external immobilization, which provides support and protection. Casting is one of the standard methods, with plaster or fiberglass cast material. A below-elbow cast is indicated for stable distal fractures, while an above-elbow cast is used when more rotation control is needed. Splints, such as sugar-tong or volar splints, are often used in the acute setting to address swelling and are usually replaced by a circumferential cast at a later time. Recently, 3D-printed casts have also been used, offering the potential for reduced weight, improved ventilation, and enhanced patient comfort.

This current research seeks to establish an inclusive in silico workflow integrating the finite element analysis with patient-specific anatomic data to validate the mechanical performance of a newly developed 3D-printed cast design applied in the immobilization of forearm fractures (11) by comparing it to a state-of-the-art solution already implemented in a clinical setting (12) and data from the literature (5). This direct biomechanical benchmarking aims to address what appears to be an under-reported area in the literature, representing a potentially valuable step for validating in-house manufactured orthopedic devices.

## 2 Materials and methods

**Figure 1:**
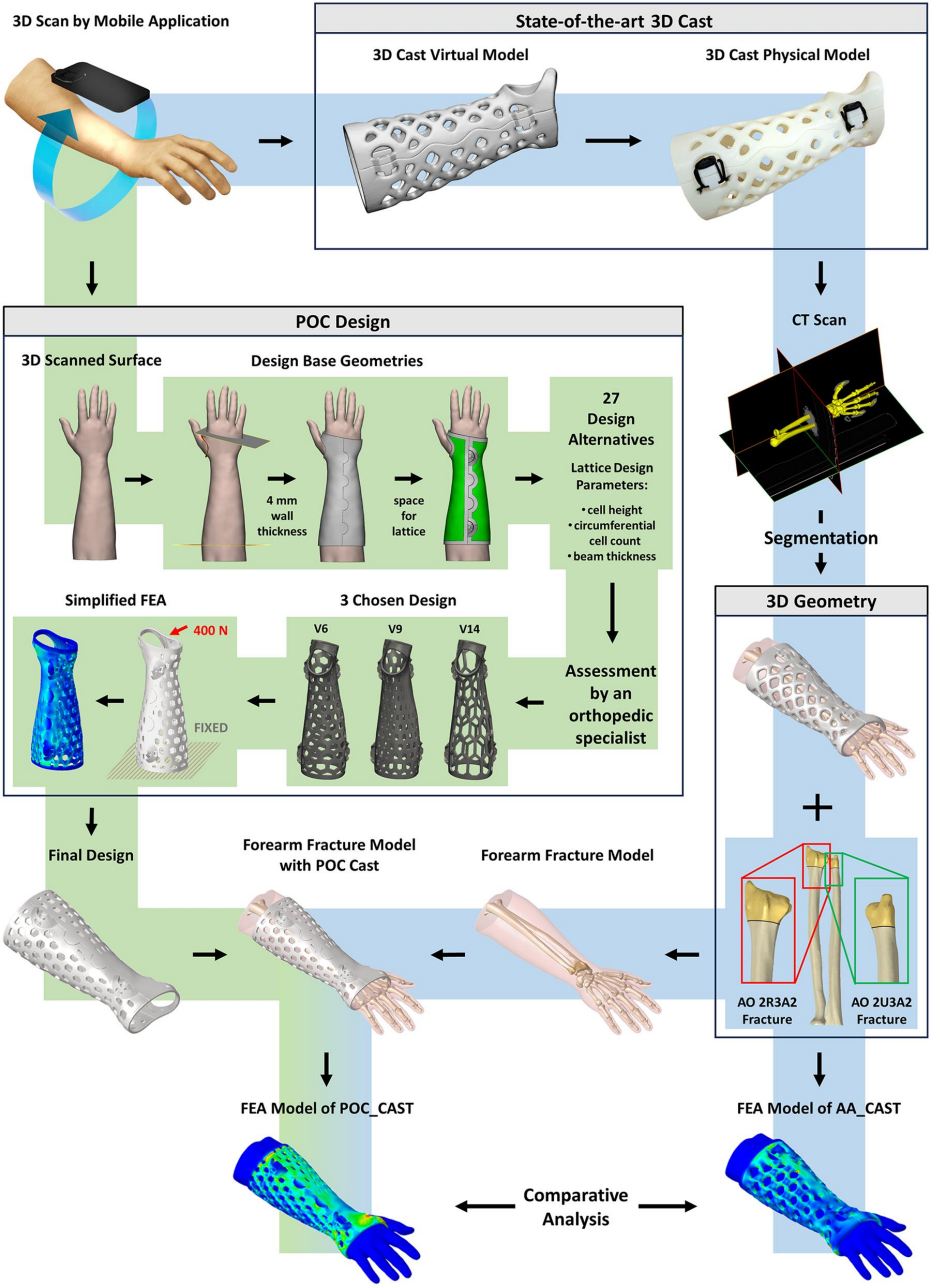
Flowchart of the in silico workflow to create and validate 3D-printed patient-specific casts for forearm fracture immobilization.

In this study, the developed in silico workflow (Figure 1) started with the creation of a patient-specific cast (AA-CAST) provided by the world-leading, state-of-the-art solution for clinically applicable 3D form cast (12)

### 2.1 3D scanning of the forearm

The forearm of a volunteer was 3D scanned using an iPhone 14, and the design order for a cock-up wrist-hand orthosis was submitted through ActivArmor’s mobile application (ActivArmor Pro).

### 2.2 Acquiring the virtual model of the AA-cast

Based on the 3D scan, the design of the 3D Cast was created by ActivArmor within a day. Both the design and 3D scan were sent back in STL file format. The STL file of the 3D scan was additionally utilized in the design of a Point-of-Care (POC) 3D Lab cast alternative (POC_CAST), presented in a subsequent section.

### 2.3 3D printing of the AA-cast design

The 3D printing of the ActivArmor cast design was carried out using Fused Deposition Modelling (FDM) technology from medical-grade ABS (13). The 3D Cast was 3D printed with a Raise3D Pro3 3D printer using print parameters recommended by ActivArmor to ensure excellent surface quality.

### 2.4 Development of the forearm finite element model with AO-2R3A2 and AO-2U3A2 fracture

The FE model of the right forearm was independently developed in our laboratory based on CT scans of a healthy 38-year-old male volunteer wearing the previously 3D-printed cast, thereby ensuring the correct hand position during imaging. The CT data were acquired in DICOM format from the hospital's PACS. The 2D CT scan images were processed in Mimics 24 software (Materialise, Leuven, Belgium) using threshold-based segmentation to reconstruct the 3D geometry of the forearm. Separate surface models of the skin, ulna, radius, carpal, and metacarpal bones were reconstructed.

The triangulated surface meshes were exported in STL format and imported into 3-Matic 18 software (Materialise, Leuven, Belgium). Surface mesh assemblies of the bones and the skin were created in this environment. Simple distal fractures of the radius (2R3A2) and the ulna (2U3A2) were modeled according to the AO Fracture and Dislocation Classification by applying planar cuts at approximately 25–30 mm from the distal articular surface (Figure 1). These selected fracture patterns represent simple, stable extra-articular fractures, which are the primary indication for conservative treatment with external immobilization.

The geometry was subsequently transferred into the HyperWorks software (Altair Engineering, Troy, Michigan, United States), where the final model was compiled. The soft tissues were incorporated through Boolean operations of the skin and bones to complete the forearm model. Volume meshes consisting of linear tetrahedral elements were generated, with the bones remeshed with an edge length of 1 mm and the soft tissues with 3 mm (5). While the model geometry is patient-specific, the material properties and boundary conditions were derived from established literature (5) to ensure comparability with previous studies. Table 1 shows the material properties used in the FE model of the forearm. Fixed connections were applied at the soft tissue–bone interfaces, while the fracture surfaces were modeled as frictionless contacts (5).

**Table 1:** Summary of the material properties, including bone and soft tissues, the different cast materials, and their associated printing technologies.

Material	Young's modulus [MPa]	Poisson’s ratio [-]	Associated printing technology	Reference
Bone	13 400	0.3	-	(5)
Soft Tissues	0.15	0.45	-	(5)
ABS	2450	0.35	FDM	(13)
Resin	2080	0.35	SLA	(38)
PA	1950	0.35	SLS	(39)

### 2.5 Development of the AA-cast finite element model

Geometries of the AA-Cast were reconstructed similarly to the bones and soft tissues of the hand in Mimics and 3-matic software and were added to the finite element model of the forearm in Hyperworks. The cast remeshed with an edge length of 1 mm and material properties of medical ABS were used (Table 1) in accordance with the manufacturer's specifications for 3D printing using FDM technology. Fixed connections were applied at the cast–soft tissue and between the two parts of the cast. (5). Six loading conditions were simulated to assess the fracture stabilization performance of the cast: flexion (FLE), extension (EXT), radial deviation (RDE), ulnar deviation (UDE), pronation (PRO), and supination (SUP). The proximal end of the model was fully constrained, and loads were applied at the distal end. For PRO and SUP, a 1 Nm moment was applied to the dorsal aspect of the cast, whereas for FLE, EXT, RDE, and UDE, a 400 N compression load was applied at the dorsal, ventral, and medial aspects of the cast's distal end, respectively (5). These loading magnitudes represent high-load scenarios that approximate worst-case daily activities rather than resting loads, thereby providing a conservative safety margin for assessing the cast's structural integrity. Furthermore, this approach is also in agreement with other recent in silico biomechanical evaluations of 3D-printed forearm casts (14, 15). The FE simulations were solved in Abaqus Standard (v2023, Dassault Systèmes, Vélizy-Villacoublay, France). The maximum von Mises stress in the cast and at the fracture surface, as well as the relative fracture displacements, were evaluated.

### 2.6 Creating POC cast designs

Taking the original 3D scan and ActivArmor's cast design as a reference for the geometric bounding volume and the size of the thumb cutout, a base geometry was created with 4 mm wall thickness in accordance with previous findings in the literature (16–18). This geometry was split into two halves, which were further divided into 10 mm wide 3D frames and the starting geometries for lattice creation in 3-Matic 18 software (Materialise, Leuven, Belgium) (Figure 1). Subsequently, the geometries were exported in STL format into NTop (nTopology, New York, USA) software. As a previous study (19) showed, superior mechanical properties can be achieved by applying hexagonal cutouts for ventilation holes. Therefore, a hexagonal shape was used as a basic element for creating the fill pattern for the areas within the frame in NTop. Twenty-seven different alternatives were created based on cell height, circumferential cell count, and beam thickness (Supplementary Figure 1-27). These design variations were assessed by an orthopedic specialist, and three versions were selected (POC_DESIGN_V6, POC_DESIGN_V9, POC_DESIGN_V14) (Figure 1) based on structural characteristics. A simplified FEA model (Figure 1) of only the cast geometry was developed in HyperWorks and Abaqus Standard for all three versions with ABS material properties. A force of 400 N was applied to the distal end of the cast while the proximal end was fixed. The best performing version was chosen based on the maximum stress and displacement results of these simplified FE models.

### 2.7 Development of the PoC-cast element model

The POC_CAST finite element model was developed by incorporating the final POC Cast design into the previously established forearm model, with all other components and boundary conditions maintained identical to those used for the AA-Cast simulations. In addition to ActivArmor's recommended FDM production method with medical ABS, two other 3D printing technologies were identified as possible options for manufacturing a 3D Cast at a Point-of-Care 3D lab. Therefore, further tests were performed on the POC Cast design for two additional materials: Formlabs BioMed Clear (Resin), used by the Formlabs Form4B Stereolithography (SLA) 3D printer, and White Nylon Medical (PA), used by the Formlabs Fuse 1+ Selective Laser Sintering (SLS) 3D printer. Table 1 shows the additional material properties used in the finite element model of the POC_CAST.

## 3 Results

**Figure 2:**
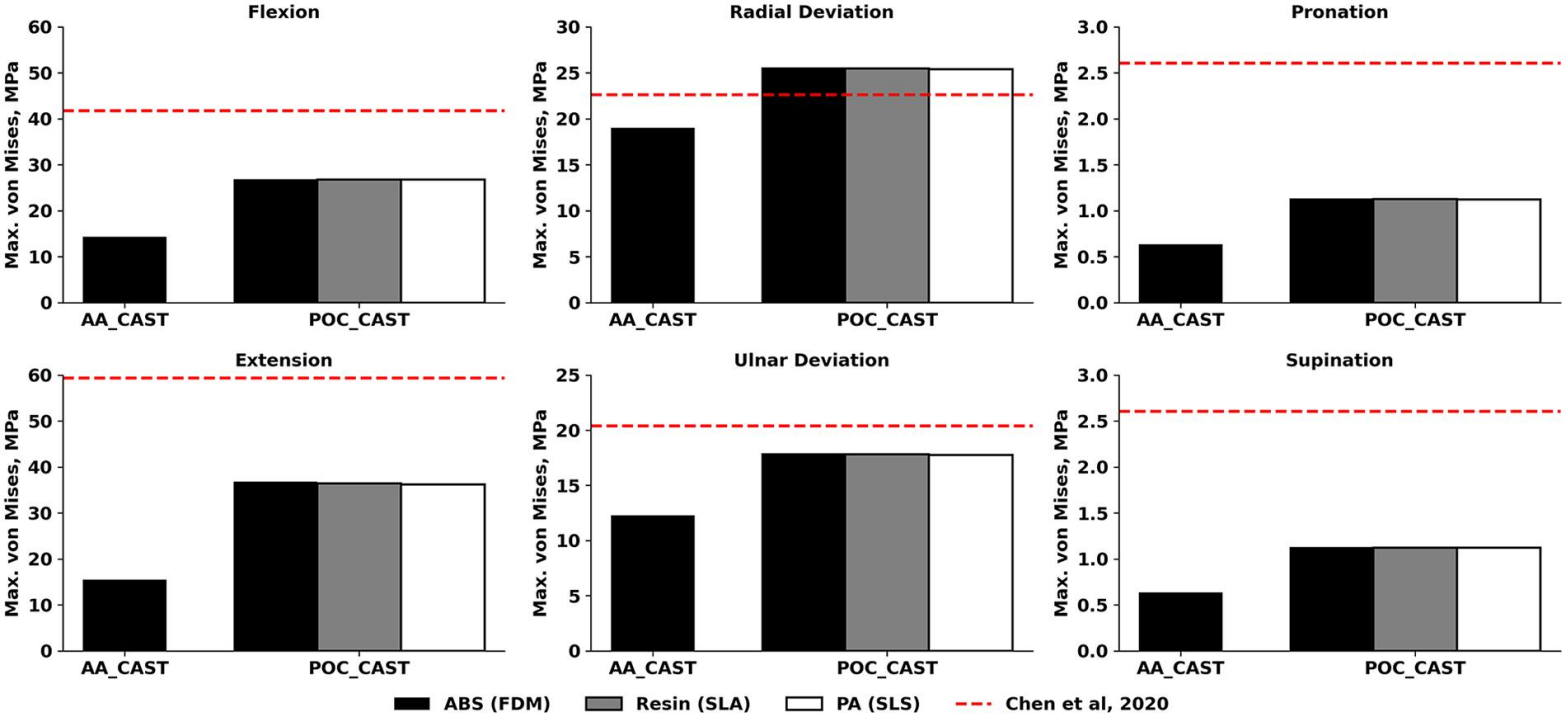
Maximum von Mises stress values in the casts for different 3D-printing technologies. Cast stresses were compared to the in silico results of Chen et al. (5). ABS, Acrylonitrile Butadiene Styrene; PA, polyamide; FDM, fused deposition modeling; SLA, stereolithography; SLS, selective laser sintering.

**Figure 3:**
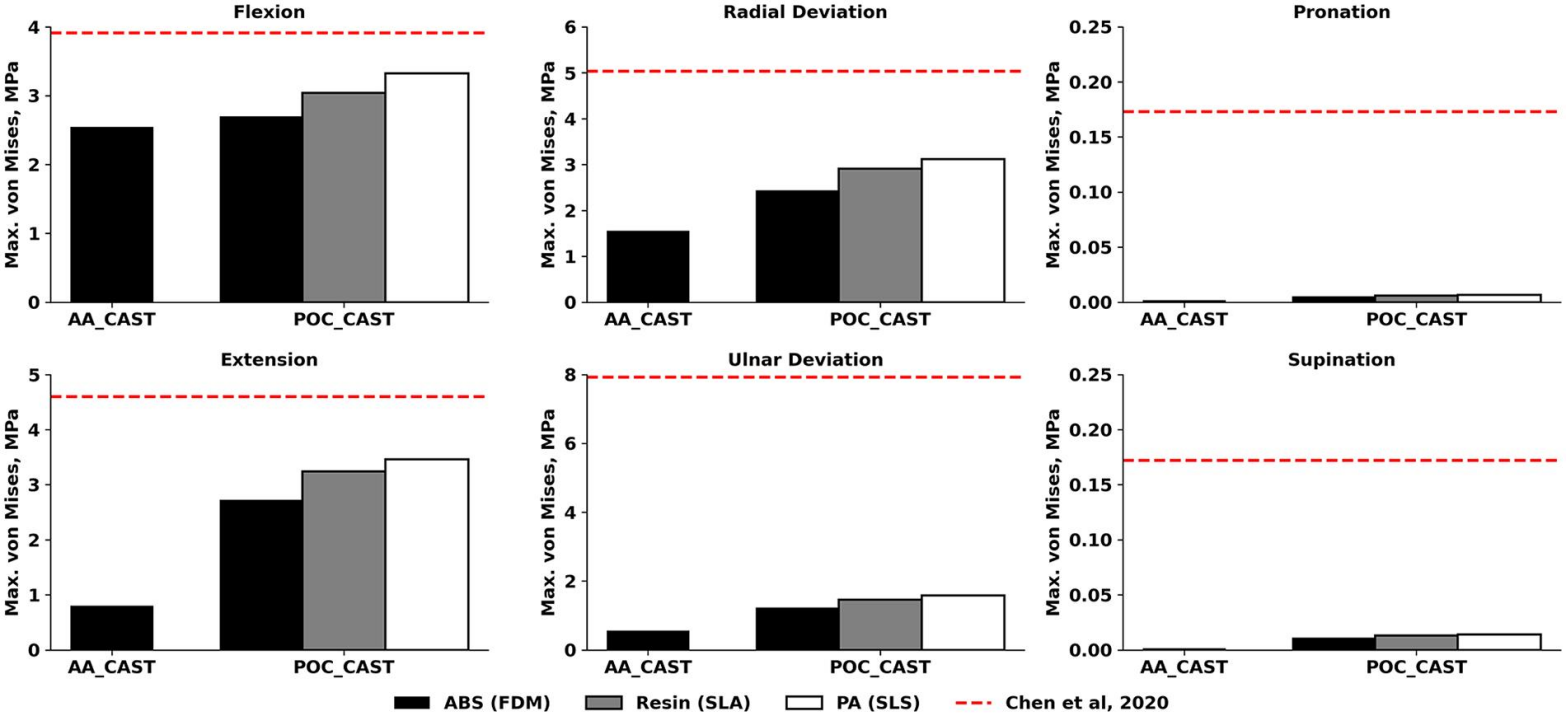
Maximum von Mises stress values in the fracture surfaces for different 3D-printing technologies. Fracture stresses were compared to the in silico results of Chen et al. (5). ABS, Acrylonitrile Butadiene Styrene; PA, polyamide; FDM, fused deposition modeling; SLA, stereolithography; SLS, selective laser sintering.

**Figure 4:**
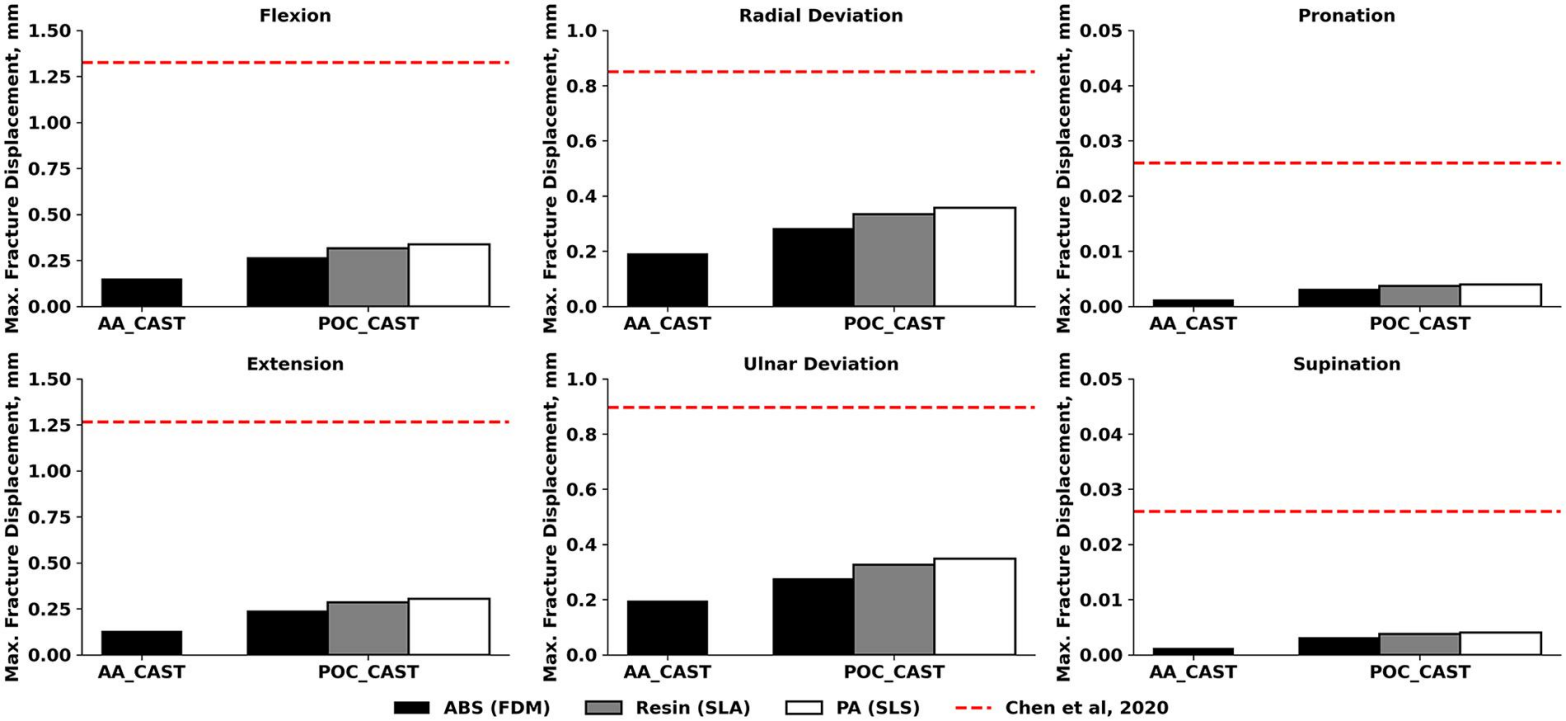
Maximum displacement of the fracture surfaces for different 3D-printing technologies. Fracture displacements were compared to the in silico results of Chen et al. (5). ABS, Acrylonitrile Butadiene Styrene; PA, polyamide; FDM, fused deposition modeling; SLA, stereolithography; SLS, selective laser sintering.

The present study evaluated the mechanical performance of two cast designs for forearm fracture immobilization under six physiological load cases, using a finite element model of the forearm. The analysis focused on the maximum von Mises stress in the cast (Figure 2), stresses in the fracture surfaces (Figure 3), and the maximum displacement of the fracture surfaces (Figure 4). This comprehensive evaluation was preceded by an initial simplified finite element study, in which three out of twenty-seven cast designs were assessed under extension only, based on maximum von Mises stress and maximum displacement within the cast.

### 3.1 Simplified FEA of the three chosen POC designs

The highest maximum von Mises stress in the cast occurred in POC_CAST_V14 (63.7 MPA), followed by POC_CAST_V6 (34.7 MPA), and POC_CAST_V9 had the lowest maximum (33.5 MPA). Similarly, for maximum displacement, POC_CAST_V14 exhibited the highest value (7.75 mm), followed by V6 (3.7 mm) and V9 (3.1 mm). Based on these results, POC_CAST_V9 (POC_CAST) was chosen as the final design for the subsequent comprehensive assessments.

### 3.2 Maximum von Mises stress in the cast

The maximum von Mises stresses in the cast showed a clear dependence on load case (Figure 2). Extension consistently produced the largest stresses, with values close to 36–37 MPa for all three materials for POC_CAST. Flexion and radial deviation followed, producing stresses in the 25–27 MPa range, while ulnar deviation generated lower values between 17 and 18 MPa. In torsional loading (pronation and supination), cast stresses were relatively low, not exceeding 1.2 MPa. In contrast, AA_CAST demonstrated lower stress levels across all loading conditions. The maximum stress occurred in radial deviation (19 MPa), followed by ulnar deviation (12.3 MPa), and flexion–extension (14,4 and 15.5 MPa, respectively), while pronation–supination remained minimal (0.6 MPa).

### 3.3 Maximum von Mises stress in the fracture surfaces

The maximum von Mises stresses at the fracture surfaces were substantially lower than those observed in the cast (Figure 3). For POC_CAST, the highest stresses were observed in extension (3.46 MPa for PA, 3.24 MPa for Resin, and 2.73 MPa for ABS), followed closely by flexion (3.32 MPa for PA, 3.04 MPa for Resin, and 2.70 MPa for ABS). Radial deviation produced similar stresses to flexion (3.11 MPa for PA, 2.91 MPa for Resin, and 2.44 MPa for ABS). In contrast, ulnar deviation resulted in lower stresses (1.59 MPa for PA, 1.46 MPa for Resin, and 1.24 MPa for ABS). Pronation and supination led to minimal stresses, not exceeding 0.014 MPa in any POC_CAST design. In the AA_CAST, stresses were consistently lower, with 2.55 MPa in flexion, 1.56 MPa in radial deviation, 0.80 MPa in extension, 0.57 MPa in ulnar deviation, and less than 0.002 MPa in pronation–supination.

### 3.4 Maximum displacement of the fracture surfaces

The maximum fracture displacements followed a similar load-dependent pattern but were generally small in absolute magnitude (Figure 4). For POC_CAST, the largest separations occurred in radial deviation (0.36 mm for PA, 0.33 mm for Resin, and 0.28 mm for ABS), followed by ulnar deviation (0.35 mm for PA, 0.33 mm for Resin, and 0.28 mm for ABS), flexion (0.34 mm for PA, 0.32 mm for Resin, and 0.27 mm for ABS), and extension (0.30 mm for PA, 0.28 mm for Resin, and 0.24 mm for ABS). Pronation and supination, in contrast, resulted in negligible displacements, not exceeding 0.004 mm across any POC_CAST design. In the AA_CAST, displacements were consistently lower, with 0.20 mm in ulnar deviation, 0.19 mm in radial deviation, 0.15 mm in flexion, 0.13 mm in extension, and approximately 0.001 mm in pronation–supination.

## 4 Discussion

Forearm fractures are among the most common orthopedic injuries (9, 20), and their management typically relies on conventional immobilization techniques such as plaster or fiberglass casts. The primary drawbacks of traditional immobilization methods include discomfort for patients, insufficient breathability, and a high risk of skin irritation (4). Advances in 3D scanning and 3D printing technologies have led to a growing interest in using 3D-printed casts at point-of-care settings to address common cast application issues, such as patient comfort and hygiene, while enabling faster and more sustainable production methods using open-lattice, lightweight designs (18, 21, 22).

Despite the growing commercial availability of 3D-printed immobilization devices, established procedures for systematic design validation and clinical implementation are missing (23). Most existing products are developed by specialized companies, with design centralized rather than integrated into the clinical environment. This can lead to issues of inaccessibility and scalability, particularly in healthcare systems with resource constraints. The establishment of an in silico workflow to validate 3D-printed patient-specific casts for forearm fracture immobilization represents a possible solution for clinical integrated 3D Laboratories to implement the creation of 3D printed forearm casts in their activities. To evaluate the feasibility and robustness of this approach, we conducted a usability validation by putting our in-house design (POC_CAST) to the test in the same FEM that was created with the State-of-the-Art AA_CAST. Finite Element Analysis (FEA) was applied to both designs, simulating a forearm fracture under clinically relevant load conditions. The integration of computational modeling and simulation into the design and validation phases enables a thorough assessment of the mechanical properties and performance of the forearm casts prior to clinical application. As demonstrated in studies comparing traditional and additively manufactured splints, FEA is an effective method for evaluating novel designs and predicting their performance under real-world loads (24). This approach helps clinicians better understand the biomechanical features of injured tissues and the medical devices used to treat them (5). While a direct simulation of conventional plaster or fiberglass casts was not performed in this study, the biomechanical performance of these traditional materials has been extensively characterized in the literature (25–27). The primary objective of this work was to establish and validate a digital workflow for designing patient-specific 3D-printed casts by benchmarking a novel point-of-care design against an established, commercially available 3D-printed standard.

All stresses in the present study were below the previously reported in silico results (5), except for POC_CAST in radial deviation, which showed a 13% increase. Printing material did not substantially affect cast stress for POC_CAST, as the maximum difference between the values obtained with ABS, Resin, and PA was 0.6 MPa in extension, which is within 2% of the mean stress for that load. This observation aligns with evaluations of common printing polymers by Sala et al. (14), who performed detailed comparisons of materials like ABS, PA, and composites. The higher stress values observed in our POC_CAST designs may be attributable to stress concentrations at geometric discontinuities. It is well-established that features like ventilation holes and thumb openings can become points of high stress, a finding consistent with FEA of other custom 3D-printed splints (14, 24, 28). All maximal fracture stresses were below published in silico values (5). Printing material had a measurable but load-dependent influence on POC_CAST fracture stresses. Max differences between materials were below 0.7 MPa within all load cases. The largest observed absolute difference was 0.7 MPa in extension (between PA and ABS), equal to 23% relative to the mean stress for that load, while the largest relative difference was in ulnar deviation by 25%. By contrast, stresses on pronation and supination were close to zero, making any percentage differences inconsequential, but the trends are similar here as well. These results indicate that while material choice had little effect on the overall load hierarchy, it could shift absolute stresses by up to one-fourth under certain conditions. This finding may be clinically relevant, as it provides comparative performance data on readily available materials, potentially informing the selection process within a point-of-care 3D printing setting. The observed low fracture stresses demonstrate that both cast designs effectively absorb and redirect physiological loads away from the injury site. This successful load sharing is the primary goal of external fixation, as it provides the mechanical stability required to promote an optimal environment for bone healing (29, 30). Compared with published in silico data (5), where fracture displacements ranged from 0.026 mm in pronation-supination to 1.33 mm in flexion, the present results were substantially lower in all loading cases, and the hierarchy of the most demanding loads also differed. Printing material had a measurable effect on POC_CAST fracture displacements. The largest difference was 0.07 mm in radial deviation (between PA and ABS), equal to 23% relative to the mean displacement for that condition. Overall, while material choice did not change the order of loading demands, it could alter absolute fracture separations by up to one-fifth under specific conditions. These fracture displacement results compare favourably with previous cadaveric studies, which have shown interfragmentary displacements of approximately 0.2 mm for traditional casts and thermo-formable braces under initial load (26), and around 0.3 mm for fracture models stabilized with more rigid internal fixation plates (31). Furthermore, all maximal fracture displacements are below 1.0 mm, which provides a strong biomechanical rationale for the stability offered by all simulated casts. This threshold is clinically significant because the mechanobiology of fracture healing is highly sensitive to interfragmentary motion; foundational research has established that while small micromovements stimulate callus, excessive strain in the fracture gap inhibits bone formation and leads to the development of fibrous tissue, risking delayed or non-union (30). This principle has been directly applied as a benchmark in modern cadaveric studies of forearm casts, where maintaining motion below this 1.0 mm level is considered to represent sufficient stability for a favorable clinical outcome (25).

A critical consideration in acute fracture management is the initial post-traumatic swelling. Traditional treatment pathways often involve a two-stage approach: an initial, accommodating splint (e.g., a sugar-tong splint) for the first few days to a week, followed by a definitive circumferential cast once the swelling has largely resolved (21, 32). The 3D-printed casts evaluated in this study are ideally suited for this second, definitive phase of immobilization (18, 33). While their two-part design allows for some minor adjustments, their primary advantages—lightweight comfort, hygiene, and a precise fit—provide the most patient benefit during the subsequent weeks of healing when compliance is key (22, 32). Therefore, the clinical posture for these devices is not as an immediate solution for acutely swollen injuries, but as an alternative to the traditional plaster or fiberglass cast for definitive, long-term immobilization.

Considering the design process of the 3D Cast, ActivArmor's solution provides a streamlined workflow where the 3D printable file is delivered within a day of capturing the 3D scan. Compared to this professional service, designing the cast at the point-of-care might need more steps using expensive software that requires advanced skills (18). Even though the POC cast design process involved thorough computational analysis, the in-house design process took significantly longer than acquiring the print-ready files from ActivArmor. However, this limitation is a key area of active research, with studies demonstrating streamlined and semi-automated workflows that drastically reduce design time from hours to minutes, making point-of-care fabrication increasingly viable (16, 17, 34).

In terms of manufacturing the 3D cast, it is important to note that while the AA_CAST was printed with a fine layer thickness of 0,2 mm on the Raise3D Pro3 3D printer to achieve superior surface quality, on a 3D printer equipped with a nozzle of 0,8-1 mm diameter, printing with 0,55-0,6 mm layer thickness could result a remarkably shorter production time (14, 16). Other 3D printing technologies, such as SLA or SLS 3D printing, could positively impact manufacturing efficiency (16). Faster production cycles could result in enhanced patient experience, that may be particularly relevant in light of evidence suggesting that shorter immobilization periods (e.g., three weeks versus five or more) for stable distal radius fractures can lead to better early grip strength and patient-reported outcomes without increasing radiological complications (35). A more comfortable and hygienic device could improve patient compliance and quality of life during this critical healing window. Future research should focus on refining this methodology further and exploring the manufacturing options of FDM, SLA, and SLS with biocompatible materials that comply with the QMS systems implemented in 3D Labs required by regulatory bodies such as MDR.

The limitations of the current study are consistent with those often encountered in similar research on finite element modelling. Bone and soft tissues have been simplified as linear isotropic materials, which may not fully capture the complex, nonlinear behaviour of biological tissues. The joints and ligaments of the wrist and fingers were not considered since only simulations with a forearm cast were performed, restricting wrist movements, and with loads that did not affect the joints of the fingers. Although the finite element model of the forearm could be improved by addressing these simplifications, and it would be beneficial to incorporate more complex material properties and soft tissues into future models, we believe these analyses could provide valuable comparative data to demonstrate the biomechanical effects of different forearm casts. Additionally, the interface between the cast halves was modeled as a fixed connection rather than the elastic rubber bands used in the clinical setting. Cazon et al. (24) demonstrated that while rigid constraints overestimate stiffness compared to elastic fastening, increasing the fastener preload significantly reduces splint displacement. Therefore, our model approximates a tightly secured device, representing a best-case stability scenario while reducing computational complexity. Furthermore, fracture surfaces were modeled as frictionless contacts. While this approach represents a worst-case scenario by neglecting the natural friction and interlocking of bone fragments, it ensures that the stabilizing capacity of the cast is not overestimated. At the manufacturing step of the workflow presented in this study, only FDM 3D printing was used. Although this is the technology that has been used most frequently for cast production (4, 7, 9, 16), there may be some drawbacks caused by the basic principle of building an object with fused deposition modelling. A common problem is water getting trapped within the internal structure of the appliance due to the layers created by the melted polymer not adhering properly along the full path of the model's perimeter (36). Additionally, the relatively big layer thickness (0,2-0,6 mm) utilized by this technology produces gaps between the layers, which could be ideal for bacterial colonisation (37). However, it is worth noting that in clinical trials, patients using ventilated 3D-printed casts report significantly less skin irritation than those in conventional fiberglass casts (22, 32), suggesting the practical benefits of improved ventilation may outweigh the theoretical risks associated with FDM surface texture. Both issues can be addressed with either a surface smoothing post-processing step for the FDM 3D printed part or by 3D printing the cast with another additive manufacturing method, such as SLA or SLS. Finally, statistical analysis was not conducted due to the deterministic nature of the finite element simulations, which yield identical results for identical inputs, precluding the need for probabilistic testing.

## 5 Conclusions

This study successfully established an in silico workflow using finite element analysis to evaluate the biomechanical performance of patient-specific 3D-printed forearm casts. The workflow was applied to compare a novel, computationally designed point-of-care cast design FEA model (POC_CAST) with a commercial, state-of-the-art solution (AA_CAST) FEA model, which is widely used and has shown promising results in clinical practice. The comparison was conducted under six physiological loading conditions simulating flexion-extension, deviation, and rotation. Since direct measurements cannot be performed *in vivo* (e.g., by applying strain gauges to a clinical patient) and *in vitro* testing on cadaveric specimens is limited by the inability to apply multiple cast designs to the same specimen, the use of an in silico workflow offers a promising alternative for evaluating new cast designs against established solutions. Our simulations revealed that while the commercial AA_CAST FEA model provided superior fracture stabilization with lower overall stress and displacement, the in-house designed POC_CAST FEA model also demonstrated robust mechanical properties. Despite higher stress maximums, the POC_CAST FEA model effectively limited fracture motion to clinically acceptable levels across all tested materials (ABS, Resin, and PA). The results indicate that material choice had a greater influence on fracture displacement than on the overall stress within the cast itself, providing valuable data for material selection in a point-of-care setting.

## Data Availability

The raw data supporting the conclusions of this article will be made available by the authors, without undue reservation.
