# Supplementary material for: In silico workflow for validation of patient-specific 3D-printed casts in forearm fracture immobilization
Source: Front Surg. 2026 Mar 23;13:1765652. doi: 10.3389/fsurg.2026.1765652 (PMC13050870; doi:10.3389/fsurg.2026.1765652)
Supplement: Supplementary file 1 [file Datasheet1.pdf]

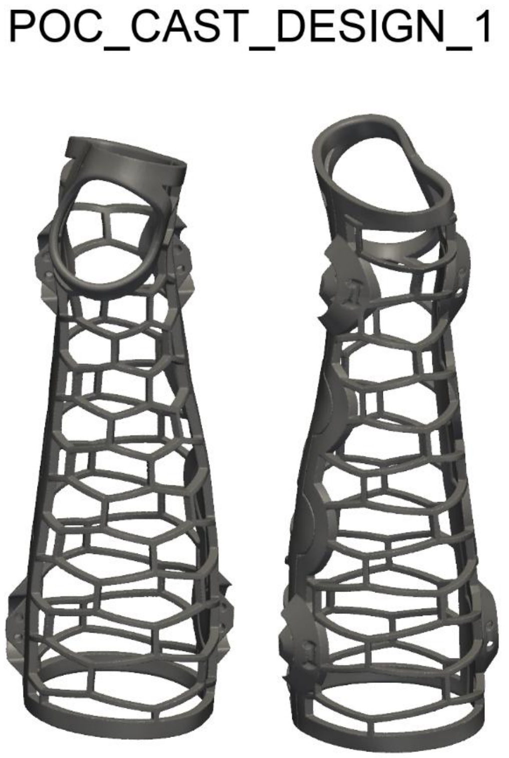

Supplementary Figure 1. POC_CAST_DESIGN_1

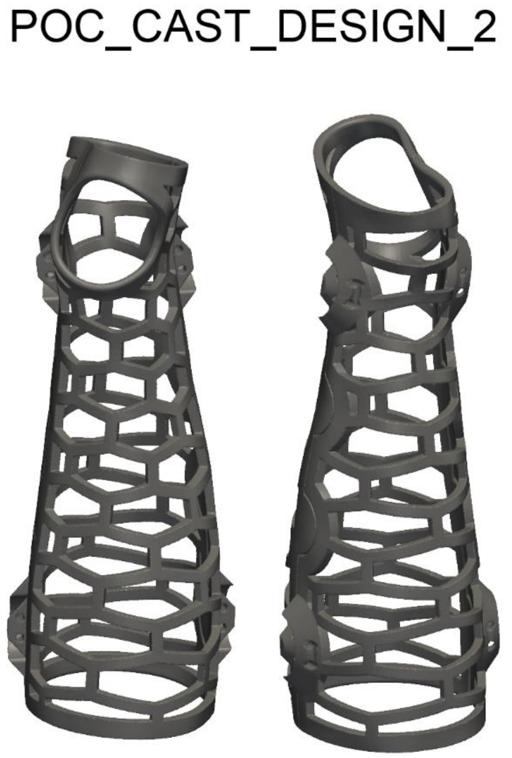

Supplementary Figure 2. POC_CAST_DESIGN_2

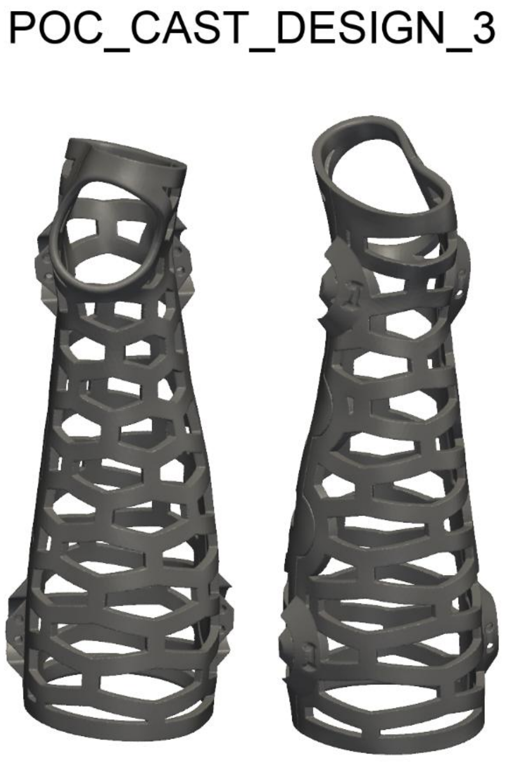

Supplementary Figure 3. POC_CAST_DESIGN_3

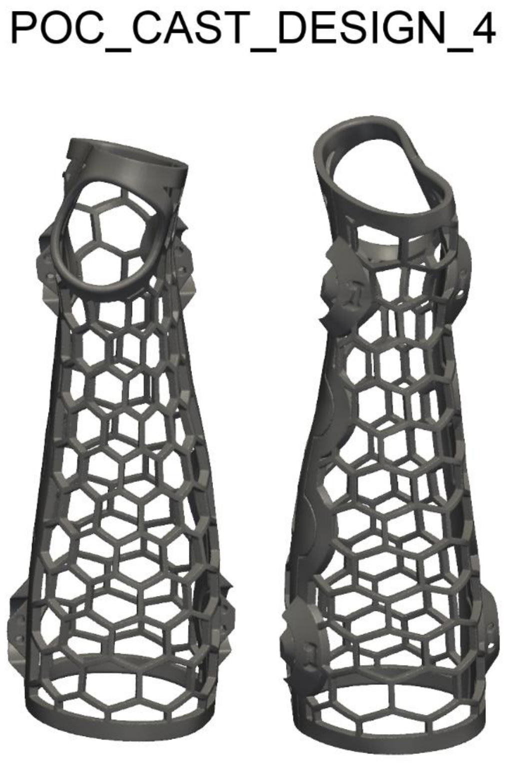

Supplementary Figure 4. POC_CAST_DESIGN_4

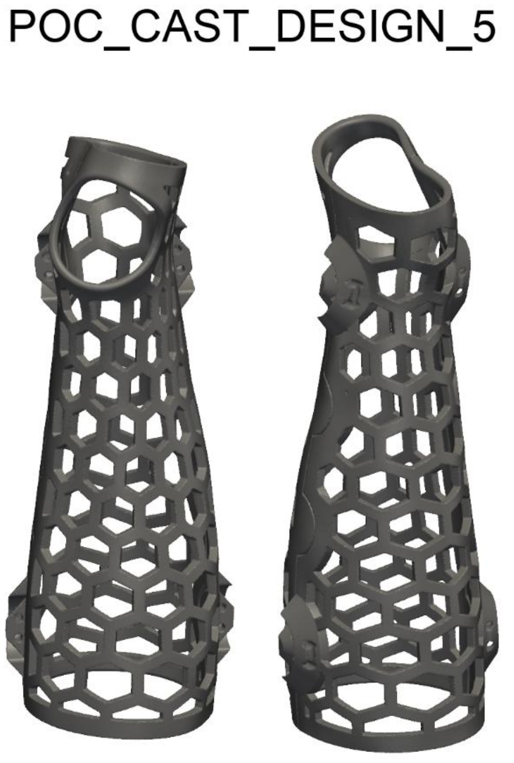

Supplementary Figure 5. POC_CAST_DESIGN_5

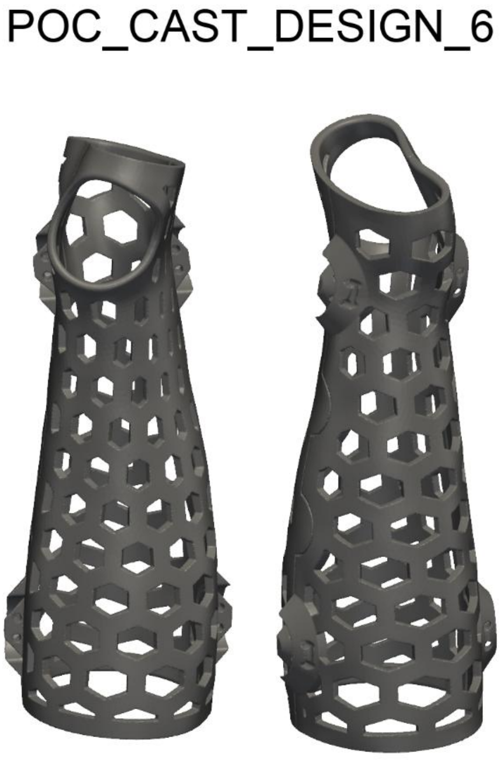

Supplementary Figure 6. POC_CAST_DESIGN_6

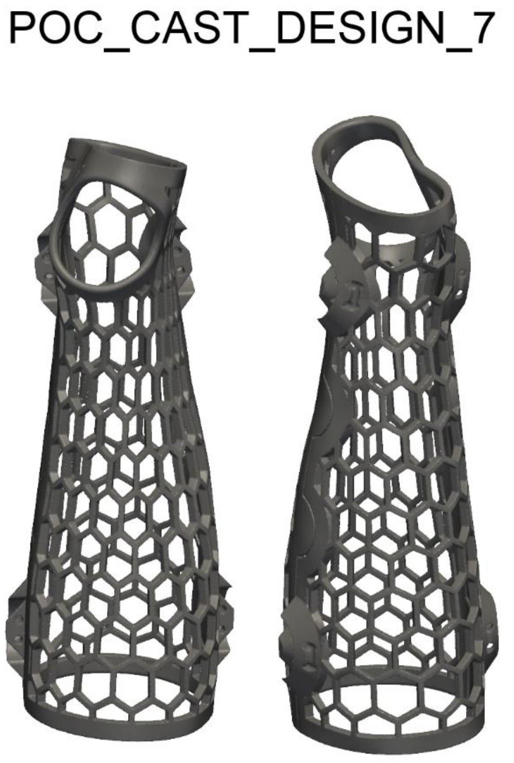

Supplementary Figure 7. POC_CAST_DESIGN_7

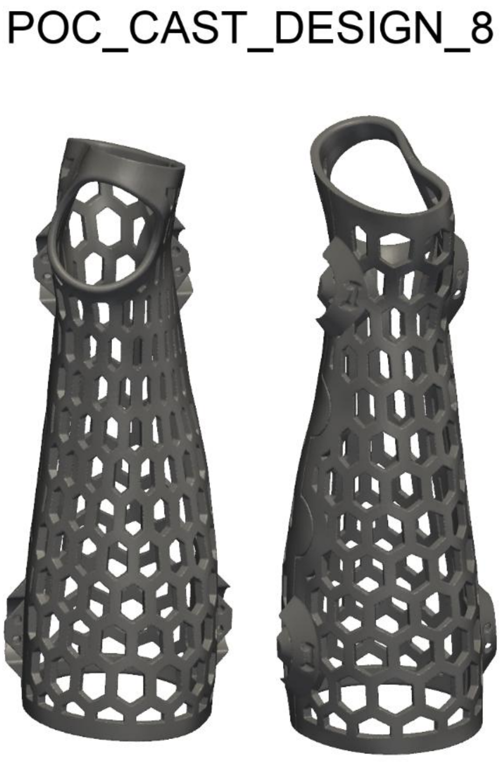

Supplementary Figure 8. POC_CAST_DESIGN_8

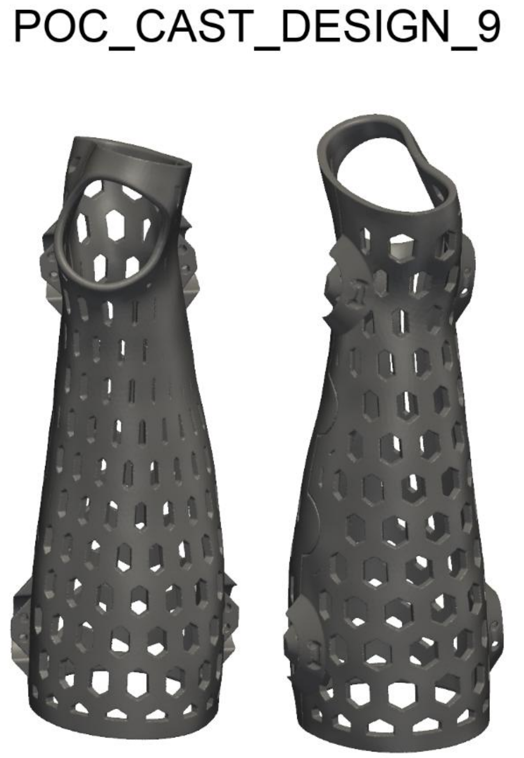

Supplementary Figure 9. POC_CAST_DESIGN_9

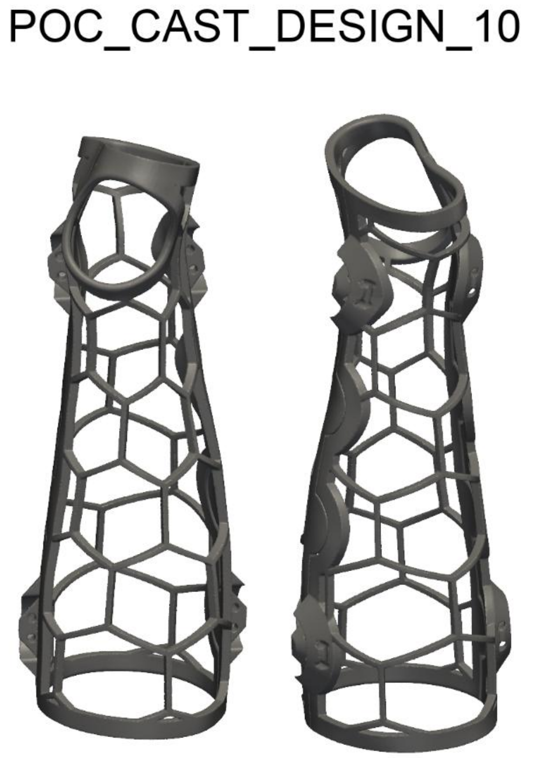

Supplementary Figure 10. POC_CAST_DESIGN_10

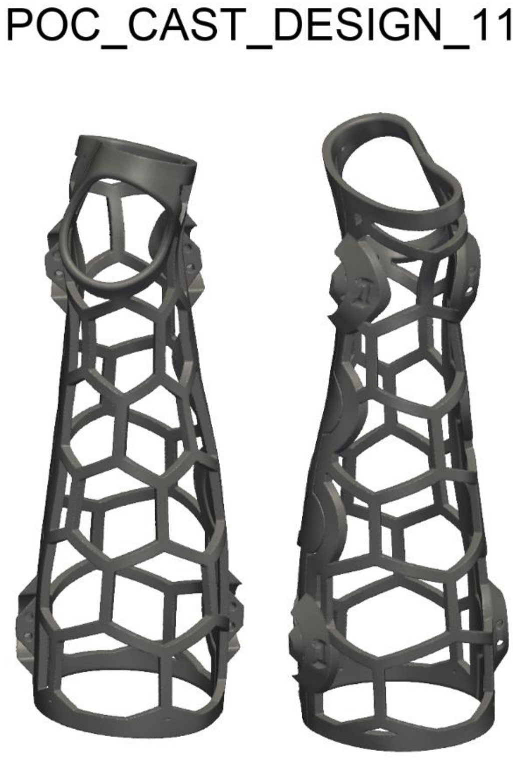

Supplementary Figure 11. POC_CAST_DESIGN_11

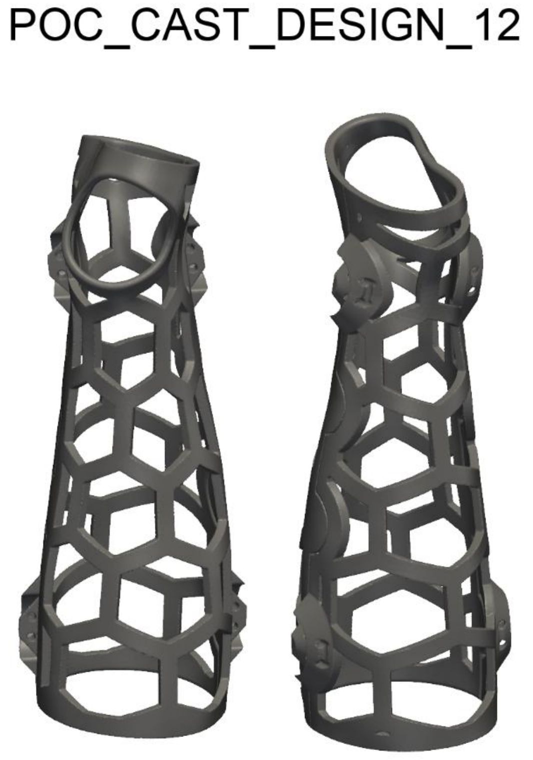

Supplementary Figure 12. POC_CAST_DESIGN_12

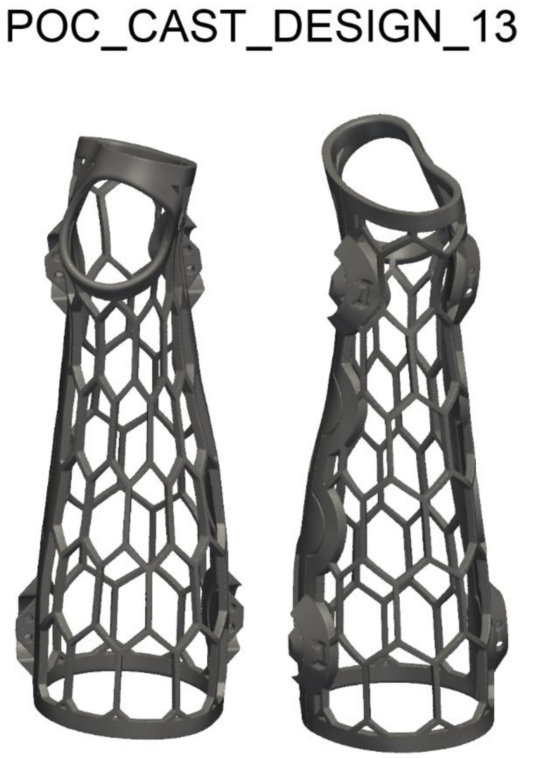

Supplementary Figure 13. POC_CAST_DESIGN_13

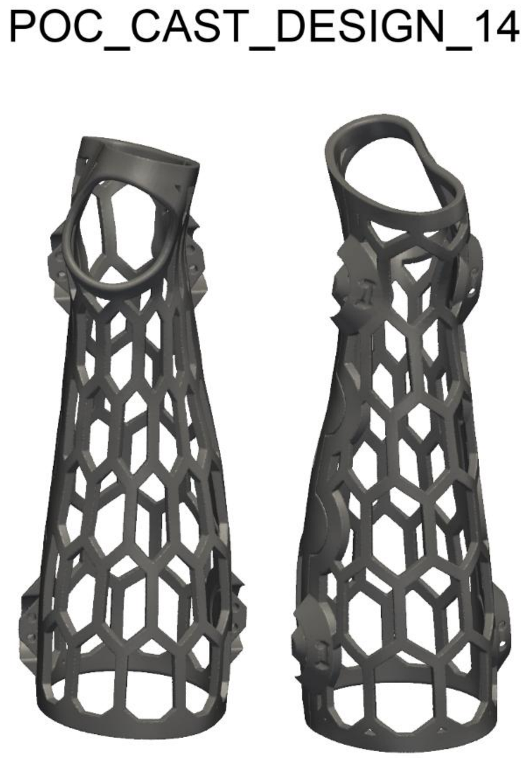

Supplementary Figure 14. POC_CAST_DESIGN_14

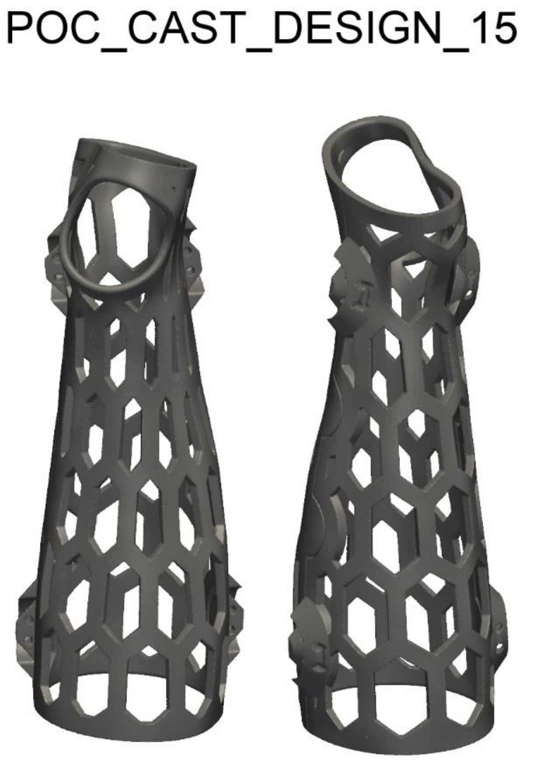

Supplementary Figure 15. POC_CAST_DESIGN_15

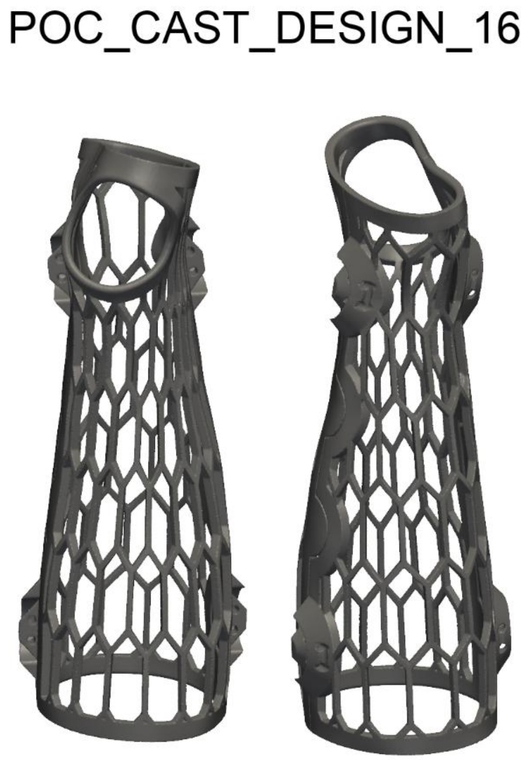

Supplementary Figure 16. POC_CAST_DESIGN_16

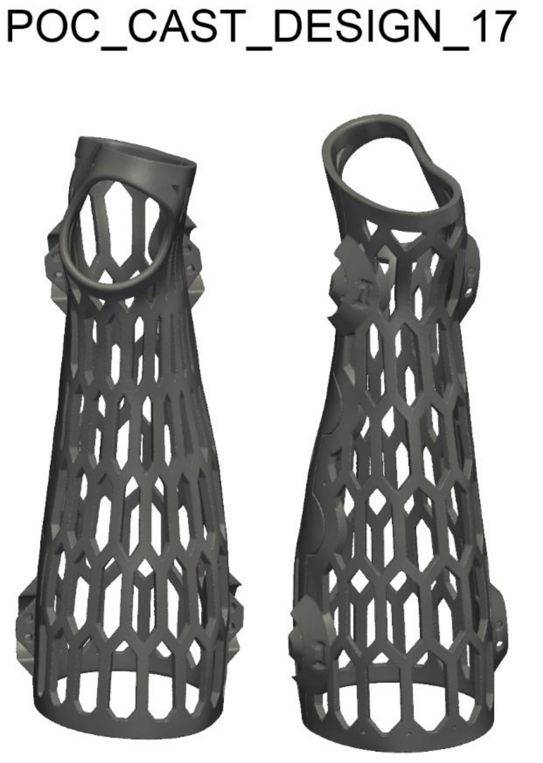

Supplementary Figure 17. POC_CAST_DESIGN_17

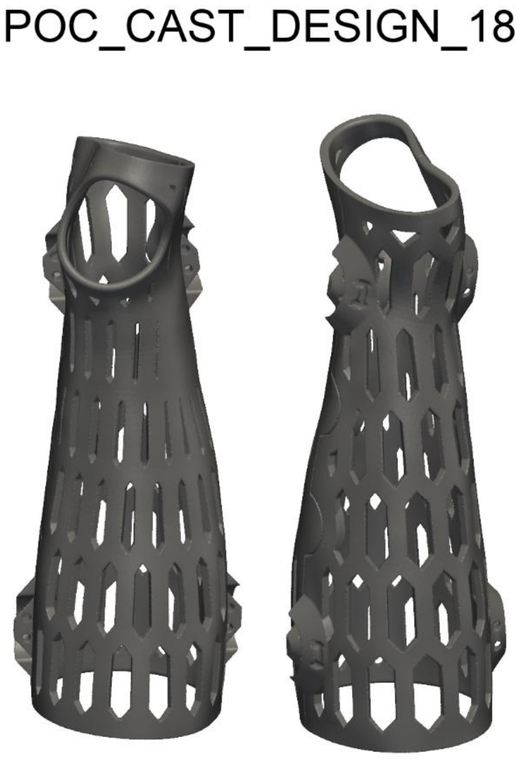

Supplementary Figure 18. POC_CAST_DESIGN_18

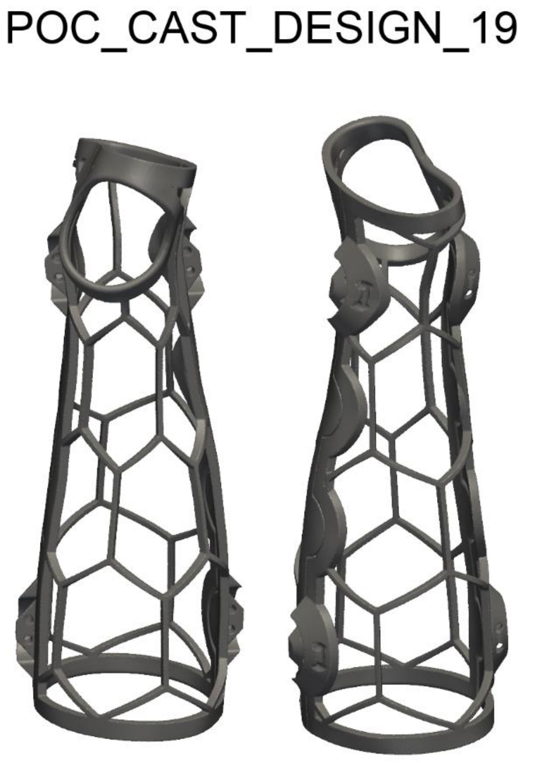

Supplementary Figure 19. POC_CAST_DESIGN_19

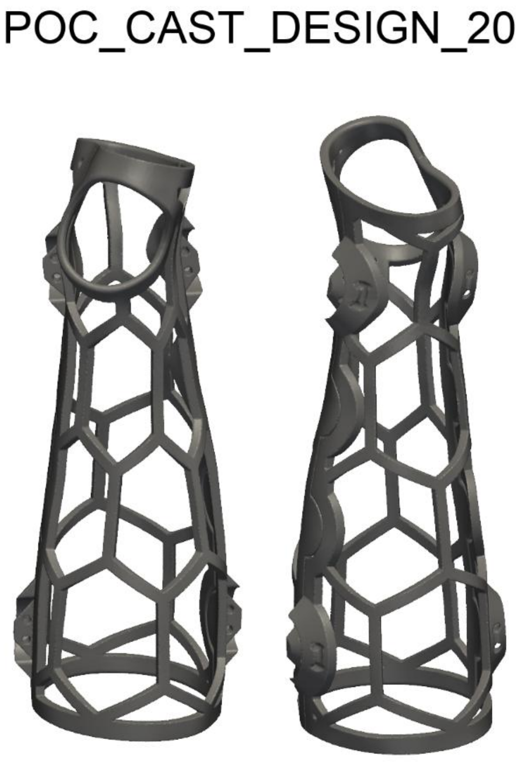

Supplementary Figure 20. POC_CAST_DESIGN_20

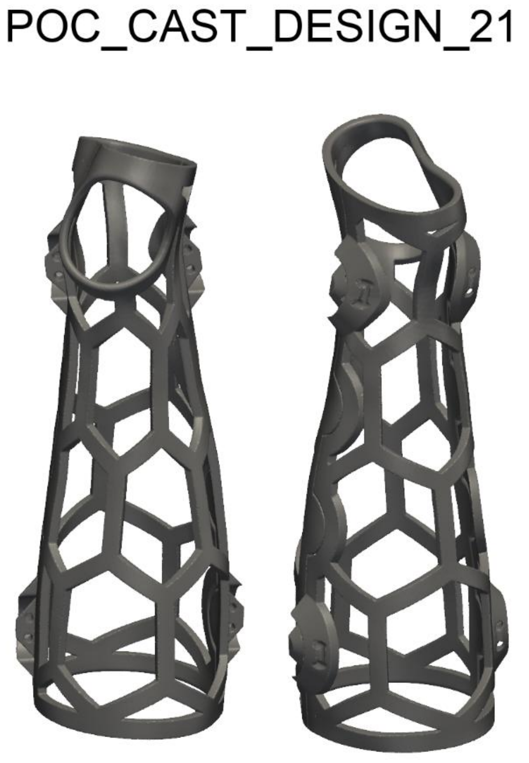

Supplementary Figure 21. POC_CAST_DESIGN_21

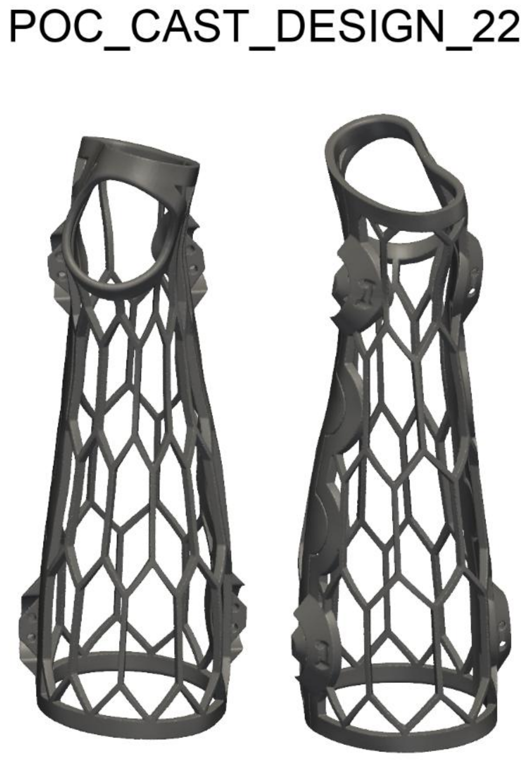

Supplementary Figure 22. POC_CAST_DESIGN_22

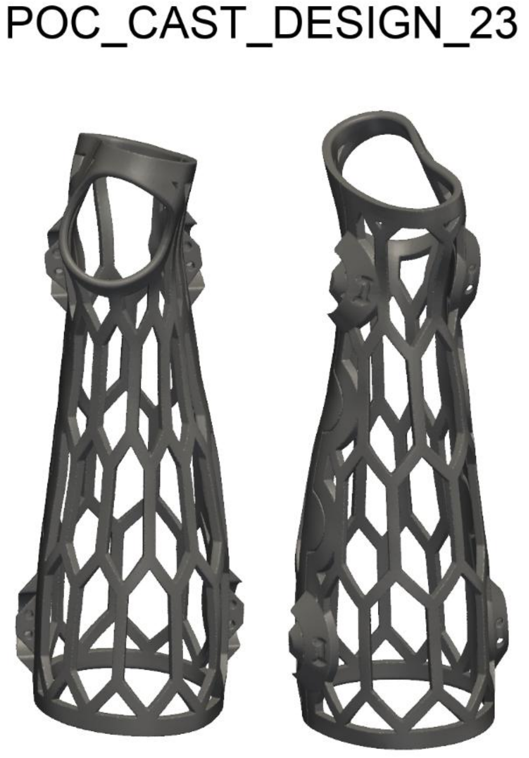

Supplementary Figure 23. POC_CAST_DESIGN_23

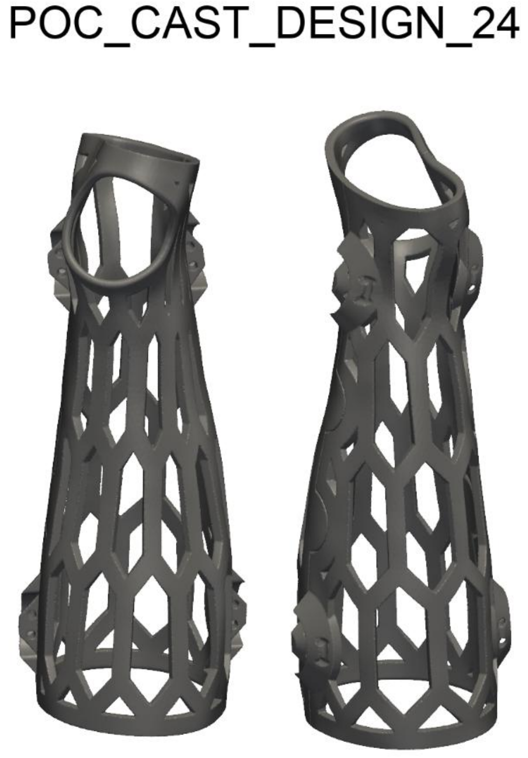

Supplementary Figure 24. POC_CAST_DESIGN_24

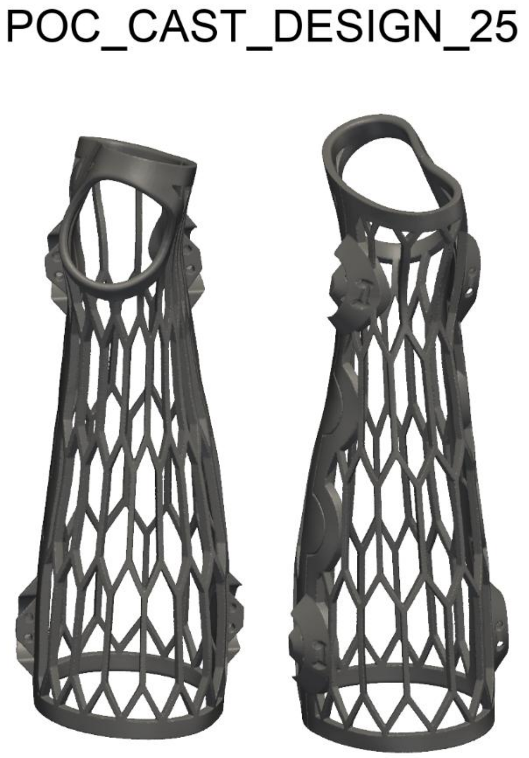

Supplementary Figure 25. POC_CAST_DESIGN_25

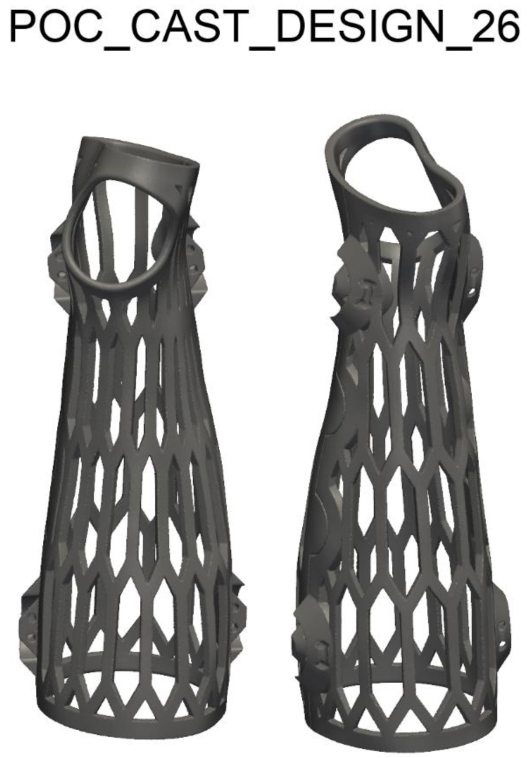

Supplementary Figure 26. POC_CAST_DESIGN_26

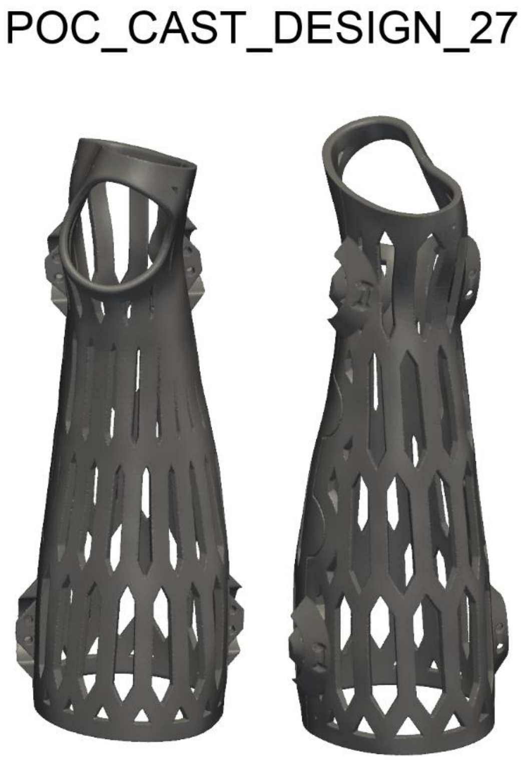

Supplementary Figure 27. POC_CAST_DESIGN_27
